# Association of schizophrenia polygenic risk score with manic and depressive psychosis in bipolar disorder

**DOI:** 10.1038/s41398-018-0242-3

**Published:** 2018-09-10

**Authors:** Matej Markota, Brandon J. Coombes, Beth R. Larrabee, Susan L. McElroy, David J. Bond, Marin Veldic, Colin L. Colby, Mohit Chauhan, Alfredo B. Cuellar-Barboza, Manuel Fuentes, Simon Kung, Miguel L. Prieto, Teresa A. Rummans, William V. Bobo, Mark A. Frye, Joanna M. Biernacka

**Affiliations:** 1 Department of Psychiatry and Psychology, Mayo Clinic, Rochester, MN USA; 2 Department of Health Sciences Research, Mayo Clinic, Rochester, MN USA; 3 Lindner Center of HOPE/University of Cincinnati, Cincinnati, OH USA; 4 Department of Psychiatry, University of Minnesota, Minneapolis, MN USA; 5 Department of Psychiatry and Psychology, Mayo Clinic, Jacksonville, FL USA; 6 Department of Psychiatry, Universidad Autónoma de Nuevo León, Monterrey, Mexico; 7 Clinica Alemana, Santiago, Chile; 8 Departamento de Psiquiatría, Facultad de Medicina, Universidad de los Andes, Santiago, Chile

## Abstract

Bipolar disorder (BD) is highly heterogeneous in symptomatology. Narrowing the clinical phenotype may increase the power to identify risk genes that contribute to particular BD subtypes. This study was designed to test the hypothesis that genetic overlap between schizophrenia (SZ) and BD is higher for BD with a history of manic psychosis. Analyses were conducted using a Mayo Clinic Bipolar Biobank cohort of 957 bipolar cases (including 333 with history of psychosis during mania, 64 with history of psychosis only during depression, 547 with no history of psychosis, and 13 with unknown history of psychosis) and 778 controls. Polygenic risk score (PRS) analysis was performed by calculating a SZ-PRS for the BD cases and controls, and comparing the calculated SZ risk between different psychosis subgroups and bipolar types. The SZ-PRS was significantly higher for BD-I cases with manic psychosis than BD-I cases with depressive psychosis (Nagelkerke’s *R*^2^ = 0.021; *p* = 0.045), BD-I cases without psychosis (*R*^2^ = 0.015; *p* = 0.007), BD-II cases without psychosis (*R*^2^ = 0.014; *p* = 0.017), and controls (*R*^2^ = 0.065; *p* = 2 × 10^−13^). No other significant differences were found. Our results show that BD-I with manic psychosis is genetically more similar to SZ than any other tested BD subgroup. Further investigations on genetics of distinct clinical phenotypes composing major psychoses may help refine the current diagnostic classification system.

## Introduction

Bipolar disorder (BD) is a highly heterogeneous illness, which presents clinical challenges and likely contributes to difficulties in identifying genetic underpinnings of this disorder^1–3^. It has been postulated that classifying BD sub-phenotypes based on clinical characteristics may help uncover underlying genetic risk factors of more genetically homogeneous disease subtypes^3,4^.

Since Kraepelin et al.^5^ described manic-depression and dementia praecox as two separate psychotic disorders, this dichotomy persisted as a part of clinical nosology^5^. However, there is accumulating evidence that these two disorders overlap in neuroimaging, neuropsychological, histological, and clinical features^6–10^. Previous studies have also consistently shown shared genetic etiology between BD and schizophrenia (SZ)^11–17^. Studies addressing the genetic overlap between BD and SZ have evolved from studying family and twin inheritance to estimating genetic correlation and performing polygenic risk score (PRS) analysis using genome-wide association data from large case–control samples. In a PRS analysis, single-nucleotide polymorphism (SNP) effect sizes from a prior genome-wide association study (GWAS) of “disease A” (e.g., SZ) can be used to calculate the estimated risk of “disease A” for a group of controls and patients with “disease B” (e.g., BD) to evaluate whether on average patients with “disease B” (i.e., BD, in our example) have elevated genetic risk of “disease A” (i.e., SZ, in our example). Evidence of this elevated genetic risk suggests the two diseases have overlapping genetic predisposition. There are at least four published PRS studies investigating the overlap between SZ and BD in the context of clinical sub-phenotypes. Ruderfer et al.^18,19^ showed that a polygenic BD signature is correlated with developing mania in SZ, and that psychotic features in BD have a significant genetic correlation with SZ. Hamshere et al.^20^ found that subjects with schizoaffective BP (SZBP) carry an excess of SZ-associated alleles compared to non-SZBP subjects. Finally, Allardayce et al.^21^ showed a gradient of SZ-PRS in the following order SZBP > BD-I with mood-incongruent psychosis > BD-I with mood congruent psychosis > BD-I without psychosis > BD-II.

In addition to growing recognition that BD with psychosis has a higher SZ-PRS compared to non-psychotic BD, there is also increasing evidence that depression and mania in BD have different genetic underpinnings^22,23^. In this study, we therefore hypothesized that psychoses found on the opposite poles of the BD spectrum, that is, manic and depressive psychoses, will differ in terms of SZ-PRS. These BD sub-phenotypes have never been studied using SZ-PRS and are important to understand as BD with psychosis carries particularly high morbidity^24,25^.

## Patients and methods

### Participants

Patients with BP were drawn from the Mayo Clinic Bipolar Biobank^26^. This biobank was established in 2009 with a primary goal to build a biorepository to study disease risk and treatment outcome^22^. Enrollment sites included Mayo Clinic, Rochester, Minnesota; Lindner Center of HOPE/University of Cincinnati College of Medicine, Cincinnati, Ohio; and the University of Minnesota, Minneapolis, Minnesota. Enrollment at each site was approved by the local Institutional Review Board, and all participants consented to use of their data for future genetic studies. Participants were identified through routine clinical appointments, from in-patients admitted in mood disorder units, and recruitment advertising. Cases were required to be between 18 and 80 years old and be able to speak English, provide informed consent, and have Diagnostic and Statistical Manual of Mental Disorders IV-TR diagnostic confirmation of BD-I or BD-II as determined by using the Structured Clinical Interview for DSM-IV (SCID)^27^. Patients who were presently psychotic or suicidal were not enrolled. Patients were assessed for a history of psychosis during manic episodes and/or depressive episodes using the SCID. In total, 1046 cases from the Bipolar Biobank were genotyped. Controls (*n* = 828) were selected from the Mayo Clinic Biobank^28^. Potential controls with International Classification of Disease-9 codes for BD or SZ in their electronic medical record were excluded. Clinical Questionnaire was used to obtain data on medications taken at the time of blood collection.

### Genotyping, imputation, and control for population stratification

The Illumina HumanOmniExpress platform was used to genotype 1046 BD cases and 828 controls (*N* = 1874; 730,499 SNPs). For quality control purposes, we excluded subjects with <98% call rate and related subjects (by including only one subject from each pair with estimated identical-by-descent allele sharing >0.2), and SNPs with call rate <98%, minor allele frequency (MAF) <0.01, and SNPs not in Hardy–Weinberg equilibrium (*P* < 1e−06). After these steps, 643,011 SNPs and 1478 subjects remained. We also performed principal component (PC) analysis in this subset; subsequent PRS analyses were adjusted for four PCs that were associated with the case–control status.

SNP imputation was performed using IMPUTE2.2.2^29^ with the 1000 genome project reference data (phase 1 data, all populations). SNPs with dosage *R*^2^ < 0.3 (poor quality imputation), and those with MAF < 0.01 (rare alleles) were removed, resulting in 7,999,324 SNPs that were included in the analysis. After quality control of genetic and clinical data, the genetic analyses included 957 cases (696 BD-I, 261 BD-II patients) and 778 controls.

### Phenotype definition

Patients were assessed for a history of psychosis during manic episodes or psychosis during depressive episodes using the SCID. Of the 957 cases, 333 (by definition BD-I) had a history of manic psychosis, 64 (42 BD-I; 22 BD-II) had a history of psychosis only during depression, 547 (312 BD-I; 235 BD-II) cases had no history of psychosis, and 13 (9 BD-I; 4 BD-II) had insufficient information to reliably determine their history of psychosis. Only 40 cases had psychosis during both mania and depression and were assigned to the manic psychosis group for the analysis. These cases were also analyzed as a separate group in a supplementary analysis.

### Polygenic risk score

We constructed polygenic risk scores (PRS)^12^ in our sample using the PRSice software^30^ based on common SNP risk effects derived from summary statistics from a large SZ GWAS conducted by the Psychiatric Genomics Consortium (PGC-SZ)^31^. In order to account for linkage disequilibrium (LD) among SNPs, clumping was performed in PLINK v1.90b3v^32^ to select relatively independent SNPs (clump-r2 was set to 0.1, and the block size threshold clump-kb to 250). In the comparison of all BD cases vs. controls, we evaluated a series of PGC-SZ association *p* value thresholds from 0.0005 to 0.5 by increments of 0.0005. A *p* value threshold of 0.039 performed best in our sample in the BD case–control comparisons, and was used subsequently for all other subgroup comparisons. The PRS for SZ (SZ-PRS) was standardized using its mean in the BD cases with no psychosis and the standard deviation (SD) in all subjects so that the cases without psychosis serve as a reference group for all comparisons. Therefore, the effect sizes in the linear regression are standardized and can be interpreted as a mean difference in standardized scores.

### Statistical analysis

We compared the mean SZ-PRS of different subgroups using linear regression with the risk score as the outcome and subgroup indicator variables as predictors. In addition, to calculate Nagelkerke’s *R*^2^, a commonly reported measure of effect size in PRS analyses, we also used logistic regression with the sub-phenotype of interest as the outcome. All analyses were performed in R (version 3.2) and were adjusted for the first four PCs to account for potential population stratification. We first compared all case subgroups described in the columns of Table 1 and the 778 controls. We next further divided our psychosis subgroups by bipolar type (BD-I or BD-II) and compared BD-I cases with manic psychosis to BD-I cases with depressive psychosis, BD-I without history of psychosis, and BD-II cases without psychosis. Other subgroups were not compared due to small sample sizes.

**Table 1:** Demographic information for cases

	All, *N* = 944	No psychosis, *N* = 547	Depressive psychosis, *N* = 64	Manic psychosis, *N* = 333
Age, mean (SD)	42.8 (15.2)	43.1 (15.7)	43.3 (15.2)	42.7 (14.5)
*Sex*				
Male	387 (40.4%)	224 (41.0%)	20 (31.2%)	138 (41.4%)
Female	570 (59.6%)	323 (59.0%)	44 (68.8%)	195 (58.6%)
*Bipolar disorder*				
Type I	696 (72.7%)	312 (57.0%)	42 (65.6%)	333 (100%)
Type II	261 (27.3%)	235 (43.0%)	22 (34.4%)	0
*Current medications*				
Lithium	304 (31.8%)	157 (28.7%)	17 (26.6%)	126 (37.8%)
Anti-psychotics	437 (45.7%)	200 (36.6%)	36 (56.2%)	196 (58.9%)
Anti-depressants	411 (43.0%)	249 (45.5%)	35 (54.7%)	124 (37.2%)
Total medications^a^, mean (SD)	1.29 (0.94)	1.19 (0.92)	1.48 (0.94)	1.44 (0.96)

*BD* bipolar disorder, *SZ* schizophrenia, *SD* standard deviation

^a^Sum of the above medications

## Results

### Sample description

Table 1 summarizes the demographic and medication information of clinically defined groups included in this study. Of the 944 cases that could be assessed for history of psychosis, 570 (60%) were female. The mean age at the time of blood draw was 42.8 years (SD = 15.2). Consistent with our phenotype definition, cases without psychosis were taking significantly less antipsychotic medications at the time of biobank enrollment than cases with depressive psychosis (*p* = 0.003) or cases with manic psychosis (*p* = 2 × 10^−10^). Cases with depressive psychosis were taking significantly more antidepressant medications than cases with manic psychosis (*p* = 0.013).

### Polygenic risk score analysis

**Fig. 1:**
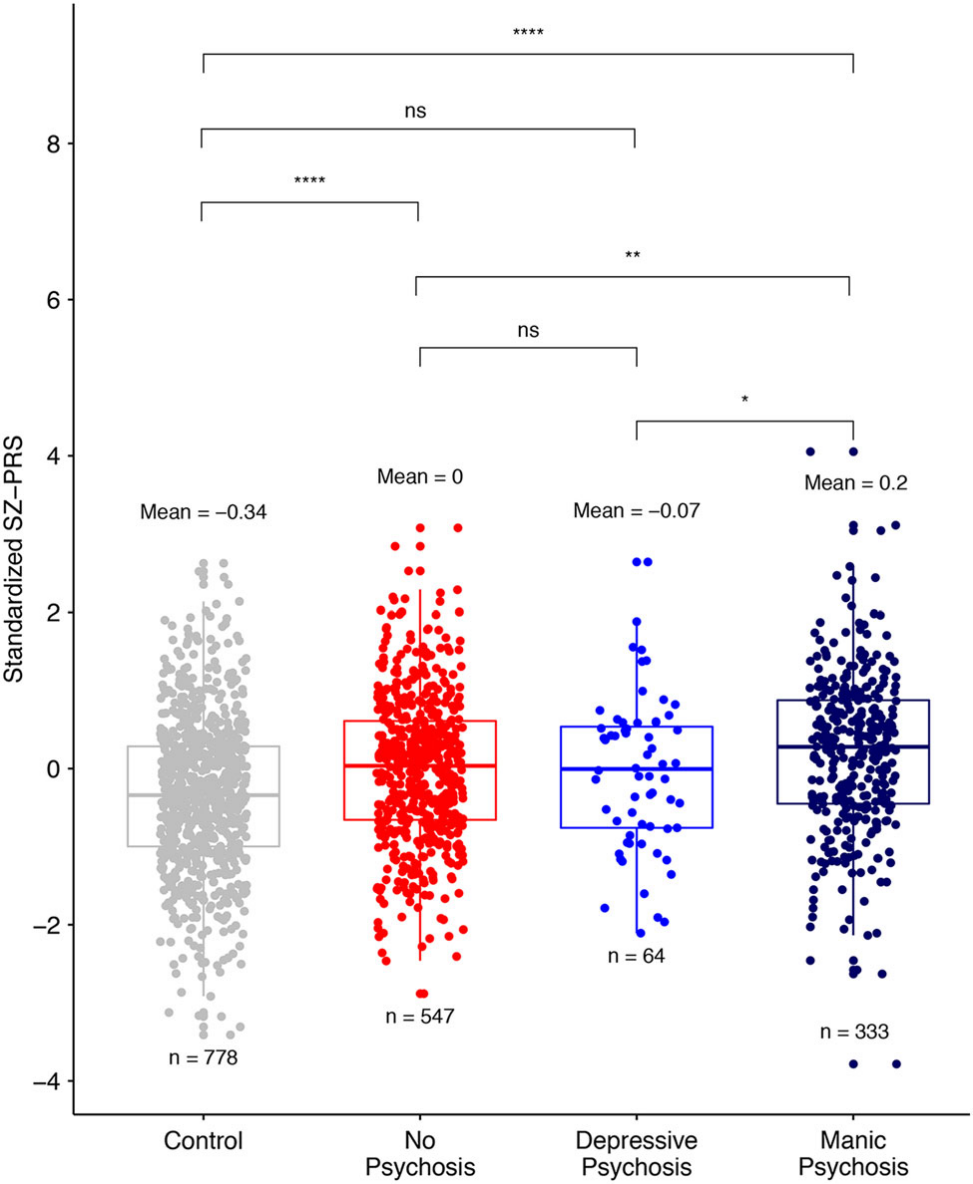
The unadjusted standardized PRS derived from PGC-SZ are plotted for controls and cases (from left to right) with no history of psychosis, psychosis during only depression, and psychosis during mania. The *y*-axis shows the standardized SZ-PRS score. The mean PRS and subgroup sample size are printed above and below each boxplot, respectively. Significance of comparisons between groups after adjustment for principal components are shown above (ns = not significant, *<0.05, **<0.01, ***<0.001, ****<0.0001)

Table 2 shows the results of the SZ-PRS comparisons between groups, as well as the proportion of variance in the phenotypes explained by the PRS (Nagelkerke’s *R*^2^). The PRS analysis comparing all Mayo BD-I and BD-II cases (*n* = 957) with controls (*n* = 778) showed evidence for association between SZ genetic risk and BD (*p* = 2 × 10^−12^). However, no difference in SZ risk between BD-I and BD-II cases was found (*p* = 0.21). Figure 1 shows the unadjusted SZ-PRS for each subgroup after stratifying BD cases into sub-phenotypes without psychosis (*n* = 547, of which 312 had BD-I and 235 had BD-II), with psychosis during only depression (*n* = 64, of which 42 had BD-I and 22 had BD-II), and with psychosis during mania (*n* = 333, consisting of only BD-I). In the PC-adjusted model comparing subgroups without psychosis, depressive psychosis, and manic psychosis to controls, the mean SZ-PRS was 0.26 (*p* = 8 × 10^−7^), 0.20 (*p* = 0.103), and 0.46 (*p* = 2 × 10^−13^) SDs higher than controls, respectively. In our case-only analysis, cases with no psychosis and cases with psychosis during mania had significantly different adjusted mean SZ-PRS (*p* = 0.0027). Cases with psychosis during mania also had significantly higher SZ-PRS than cases with psychosis during only depression (*p* = 0.043). The above conclusions did not change when the cases with psychosis during both depression and mania (*n* = 40) were analyzed separately rather than assigned to the manic psychosis group (Supplementary Figure 1).

**Table 2:** Association of polygenic risk scores across variously defined bipolar strata

	**Est (95% CI)**	***p*** **value**	**Nagelkerke’s** ***R*** ^**2**^
*Comparison to 778 controls*			
BD case (*N* = 958)	0.33 (0.24, 0.42)	2.0e−12	0.038
*Stratified by type*			
BD type I (*N* = 696)	0.35 (0.25, 0.45)	2.8e−12	0.044
BD type II (*N* = 261)	0.26 (0.13, 0.40)	9.8e−05	0.021
*Stratified by psychosis subtype*			
Manic psychosis (*N* = 333)	0.46 (0.34, 0.58)	2.1e−13	0.065
Depressive psychosis (*N* = 64)	0.20 (−0.04, 0.44)	0.103	0.007
No psychosis (*N* = 547)	0.26 (0.16, 0.37)	7.6e−07	0.025
*Within-case comparisons*			
Manic psychosis vs. no psychosis	0.20 (0.07, 0.32)	0.003	0.014
Depressive psychosis vs. no psychosis	−0.06 (−0.31, 0.18)	0.611	0.001
Manic psychosis vs. depressive psychosis	0.26 (0.01, 0.51)	0.043	0.016
*Split by BD type*			
BD-I vs. BD-II	0.09 (−0.05, 0.22)	0.209	0.003
Manic psych vs. BD-I-no psych (*N* = 312)	0.20 (0.05, 0.34)	0.007	0.015
BD-I-dep psych (*N* = 42) vs. BD-I-no psych	−0.11 (−0.41, 0.19)	0.481	0.005
Manic psychosis vs. BD-I-dep psychosis	0.31 (0.01, 0.61)	0.045	0.021
Manic psych vs. BD-II-no psych (*N* = 235)	0.19 (0.03, 0.35)	0.017	0.014
BD-I-no psych vs. BD-II-no psych	−0.01 (−0.17, 0.15)	0.922	6 × 10^−5^

*BD* bipolar disorder, *Psych* psychosis, *Est* estimated difference of standardized polygenic risk scores between the two groups, *CI* confidence interval, *Est, CI, p* value are based on linear regreesion with PRS as the outcome

**Fig. 2:**
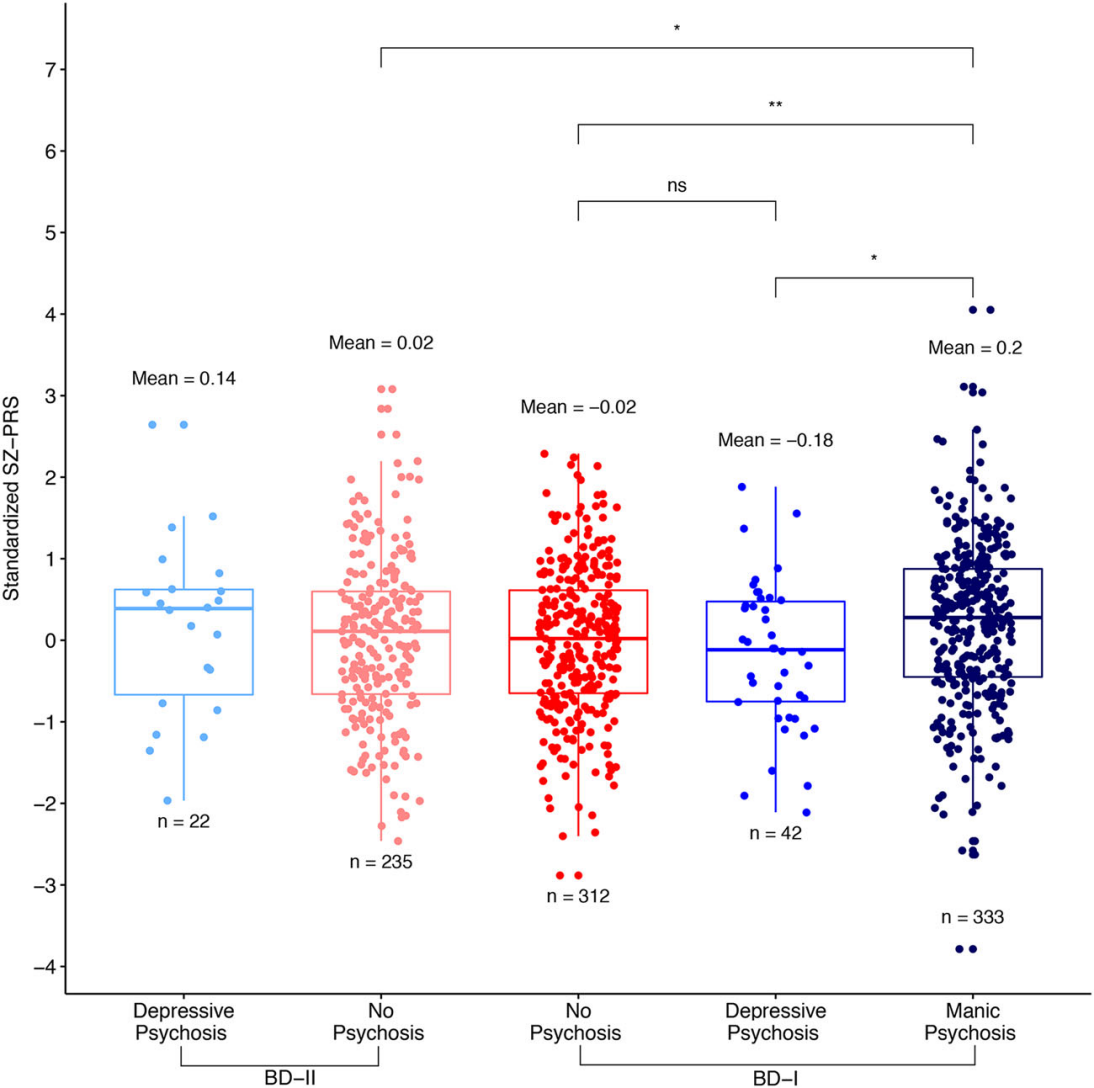
The unadjusted standardized PRS derived from PGC-SZ are plotted for BD-I and BD-II case subgroups (from left to right). The *y*-axis shows the standardized SZ-PRS score. The mean PRS and subgroup sample size are printed above and below each boxplot, respectively. Significance of comparisons between groups after adjustment for principal components are shown above (ns = not significant, *<0.05, **<0.01, ***<0.001, ****<0.0001)

Because only BD-I cases can be diagnosed with psychosis during mania, and because previous studies have shown higher SZ-PRS in BD-I than in BD-II patients^21,33^, we further divided our psychosis sub-phenotype by type of BD (Fig. 2). The differences described above increased so that the BD-I cases with psychosis during mania had 0.20 SD (*p* = 0.007) and 0.31 SD (*p* = 0.045) higher PC-adjusted mean SZ-PRS than BD-I cases without psychosis and with psychosis during depression, respectively. BD-I cases with manic psychosis also had a 0.19 SD (*p* = 0.017) higher PC-adjusted mean risk of SZ than BD-II cases without psychosis. In short, BD-I subjects with manic psychosis had 0.21 SD (*p* = 0.015) and 0.19 SD (*p* = 0.003) higher adjusted SZ risk than any other BD-I subject or BD-II subject group, respectively. There were no significant differences in SZ genetic risk between cases without psychosis and cases with psychosis during only depression in either analysis.

## Discussion

Our results show that SZ-PRS is a better predictor of BD with manic psychosis than of BD with only depressive psychosis or no history of psychosis. This suggests that BD patients with manic psychosis are genetically more similar to SZ than BD patients with a history of only depressive psychosis or BD patients with no occurrence of psychosis. Previous studies have shown higher mean SZ PRSs in BD-I than in BD-II, and in BD with psychosis than in BD without psychosis^33^. In addition, a gradient of SZ-PRS in patients with BD was shown in the following order SZBP > BD-I with mood-incongruent psychosis > BD-I with mood congruent psychosis > BD-I without psychosis > BD-II^21^. Our results are consistent with these findings and further suggest that SZ-PRS is higher in BD with manic psychosis, than in BD without psychosis or with psychosis during depression. Together with previously published PRS studies, these results further erode the traditional dichotomy between BD and SZ.

We did not find a significant difference in SZ-PRS between the BD-I and BD-II groups, which was previously shown by Charney et al^33^. However, we note that the effect size estimate for this comparison is identical in our study and in the study of Charney et al.^33^ (*R*^2^ = 0.003 in both studies), indicating our results are consistent with those of Charney et al.,^33^ and our results did not achieve statistical significance due to the smaller sample size. However, our results further suggest that the small difference in SZ-PRS observed between BD-I and BD-II cases in both our data and the data of Charney is likely largely driven by the subset of BD-I cases with psychosis during mania that compose part of the total BD-I group and have the highest SZ genetic risk scores. Our study was the first to consider BD type (I vs. II) and psychosis type (psychosis during mania vs. psychosis during depression vs. no psychosis) simultaneously, and our study suggests that SZ-PRS is not very different in BD-I without psychosis and BD-II without psychosis (Table 2). Replication of this finding in larger samples is warranted.

Our results should be interpreted in the context of the study’s limitations. First, the number of patients with depressive psychosis was relatively small leading to low power in comparisons involving this group. In particular, our study had 80% power to find differences of about 0.4 SD in the mean standardized PRS in comparisons with this group, whereas we had 80% power to detect differences as small as 0.2 SDs when comparing standardized PRSs in the manic psychosis group vs. the BD with no psychosis or control groups. Nevertheless, despite this power limitation, our study provided marginally significant evidence that SZ-PRS is lower in BD patients with psychosis during depression than in BD patients with psychosis during mania (*p* = 0.043). Age or duration of illness may also have influenced our results, since some of the young non-psychotic BD patients may potentially experience manic psychosis in the future. However, on average the manic psychosis patients were slightly younger than the patients who have not experienced psychosis, suggesting that this bias was not likely to have played an important role. Finally, multiple *p* value thresholds were used to optimize the SZ-PRS for the comparison of the full set of cases vs. controls; therefore, the threshold for statistical significance for any case–control comparison should be more stringent^30^. However, we note that the PRS was not further optimized for any within-case comparisons; thus no further adjustment for multiple testing would be required for these comparisons. Nevertheless, pairwise comparisons among multiple case subgroups were performed, which should be taken into account in interpreting the results.

Overall, our results add to the concept that SZ and BD are on a spectrum of continuously distributed genetic and phenotypic variables, rather than being two entirely discrete disorders. Our findings point to a cumulative effect of “SZ alleles” that at higher frequencies shift bipolar presentation more towards psychotic mania. These findings could help shape future diagnostic reclassification of major psychoses.

## Electronic supplementary material

supplemental figure 1
