# Supplementary material for: Association of schizophrenia polygenic risk score with manic and depressive psychosis in bipolar disorder
Source: Transl Psychiatry. 2018 Sep 10;8:188. doi: 10.1038/s41398-018-0242-3 (PMC6131184; doi:10.1038/s41398-018-0242-3)
Supplement: Supplementary file 1 — supplemental figure 1 [file 41398_2018_242_MOESM1_ESM.docx]

**Supplementary Figure 1. Boxplots of SZ-PRS scores for cases and controls with additional case group for history of both depressive and manic psychosis**


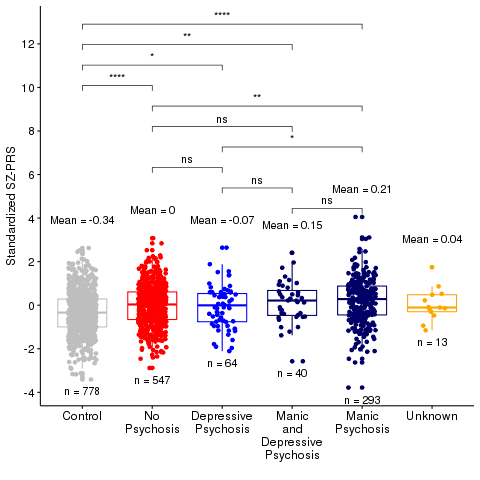


The unadjusted standardized SZ-PRS plotted for controls and four different phenotypes of cases. The mean PRS and subgroup sample size are printed above and below each boxplot, respectively. Significance of comparisons after adjustment for principal components: ns = not significant, *< 0.05, **< 0.01, ***< 0.001, ****< 0.0001.
